# 

**Published:** 1865-10

**Authors:** 


### CHICAGO MEDICAL JOURNAL.

**Terms, $2.00 per Annum.**

### CONTENTS OF THE OCTOBER NUMBER.

PAGE.

ORIGINAL COMMUNICATIONS.

Empyema. By Jas. S. Whitmire, M. D., of Metamora, Ill ..... 433

Clinical Lectures on Diseases of the Eye. By E. L. Holmes, M. D., of Chicago, Lecturer on Diseases of the Eye and Ear in Rush Medical College, &c.—The Crystalline Lens ..... 441

Pathologia Generallis. By W. H. Price ..... 444

Cerebro-Spinal Meningitis, or Spotted Fever. Treated by Blistering and Belladonna. By E. R. Travers, M. D., Amboy, Ill ..... 451

SELECTED.

History, Theory and Practice of Syphilisation. By Prof. W. Boeck, of Christiania. Delivered in the Theatre of the Meath Hospital and County of Dublin Infirmary, on Friday, June 2nd, 1865 ..... 453

New Process for Making Fluid Extracts ..... 458

Process of Disinfection ..... 459

On the Treatment of Croup by the Inhalation of Lime Water ..... 461

The Circulation of Sap in Trees, and the Formation of Maple Sugar ..... 462

Syncope—Asphyxia—Poisoning ..... 465

Prices Current ..... 467

Carbuncle ..... 469

An English Cure for Drunkenness ..... 470

Successful Surgical Operation ..... 470

Clinical Lecture on Diphtheria. By Edw. Headlam Greenhow, M. D, Physician to the Middlesex Hospital ..... 471

EDITORIAL AND MISCELLANEOUS ..... 479

